# Perceived Psychological, Economic, and Social Impact of Khat Chewing among Adolescents and Adults in Nekemte Town, East Welega Zone, West Ethiopia

**DOI:** 10.1155/2017/7427892

**Published:** 2017-02-06

**Authors:** Amsalu Taye Wondemagegn, Melese Chego Cheme, Kelemu Tilahun Kibret

**Affiliations:** Department of Public Health, College of Medical and Health Sciences, Wollega University, Nekemte, Ethiopia

## Abstract

The main aim of this study was to assess psychological, economic, and social impact of khat chewing among adolescents, in Nekemte town, East Welega Zone. A community based cross-sectional study was conducted from March to June 2016 using both quantitative and qualitative methods of data collection. A total of 359 samples were included in the study. A pretested, interviewer based structured questionnaire was employed during data collection. The study found the current prevalence of khat chewing practices was 48.6%. Perceived psychological problems especially depression and anxiety were associated with khat chewing practices. The risk of depression was about 25 times higher among khat chewers compared to nonchewers. Similarly the risk of anxiety among khat chewers was about 5 times higher compared to nonchewers. Generally current khat chewing practices in the study area are relatively high. The occurrence of reported, perceived psychological problems mainly depression and anxiety was significantly higher among khat chewers compared to nonchewers. Thus efforts like creating awareness about negative effect, making different recreation methods available, and formulating common conventions regarding khat use mainly by young generations are necessary to decrease the magnitude of chewing practices and thereby its associated consequences.

## 1. Introduction

Khat (*Catha edulis* Forssk.) is an evergreen flowering tree or shrub, first identified by Forsskal, a botanist in 1762 [1, 2]. It is mainly cultivated in East Africa and the Arabian Peninsula. Ethiopia is thought to be the origin of khat and currently it is consumed everywhere in the country mainly by youths [3]. According to EDHS 2011 the prevalence of khat use in the general population of Ethiopia was 27.3% among men and 11.0% among women of 15–49 years [4]. Similarly other studies show the prevalence of khat use in Ethiopian out school and in school adolescents was 23.0% and 7.5%, respectively [5].

A study conducted among adults in Butajira, Ethiopia, found current prevalence of khat chewing was 50% [6]. The study conducted among college students in North West Ethiopia also showed the current prevalence of khat chewing was 17.5% [7]. Similarly, institution based cross-sectional study conducted among college students in Bahir Dar town, Ethiopia, showed the overall prevalence of khat chewing in college students was 19.6% [8]. A community based study conducted in Jimma town, south west Ethiopia, found the khat chewing prevalence of 30.6% [9].

The central stimulant effects of khat are similar to those of amphetamine. The reason is that the main active ingredient in khat is psychoactive alkaloids called cathinone, an amphetamine-like substance. Chewing of khat has been practiced in many countries for social, psychological and religious reasons [10, 11]. In addition, other studies show khat is widely consumed for the purpose of elevating mood, happiness level, confidence, alertness, and thinking ability [12].

Perceived mental consequences of khat chewing practices include headaches, dizziness, impaired cognitive functioning, fine tremor, insomnia, alertness, dependence, tolerance, and anxiety [13–15]. A study conducted elsewhere revealed that, khat chewing practices were associated with perceived psychological outcomes like sleep disturbance, anxiety, depression and sedation [16]. Similarly another study also reported that khat chewing practices had been associated with many of the perceived psychological impacts [17]. Sleeping problems were reported by 65%, 51% reported loss of appetite, and 44% reported feeling the urge to chew khat.

In addition several studies have revealed that regular consumption of khat seriously affects the social and economic condition of the user [18–20]. The daily cost of khat may affect household income to fulfill nutritious food, home improvement, education or other family needs and finally leads to financial problem and family breakdown [15, 21]. Much time is spent on buying and chewing khat leaves, which affects working hours and time [22]. This causes absenteeism from work, absenteeism from class and poor academic performance of the students [23], and unemployment. Lastly death rate is significantly higher among khat chewers due to chronic illness such as heart disease and stroke as compared with non-khat-chewers [24].

Despite this impact of khat chewing practices there is only little community based epidemiological study done in Ethiopia and there has been no any previous study conducted in the current study area. Therefore, the main aim of current study was to determine the level, to identify related factors, and to show perceived psychological, economic, and social impact of khat chewing practices among adolescents and adults in Nekemte town, East Welega Zone, west Ethiopia.

## 2. Methods and Materials

### 2.1. Study Design, Study Period, and Study Area

A community based cross-sectional study was conducted from March to June 2016 using both quantitative and qualitative methods of data collection. This study was conducted in Nekemte town. Nekemte town is the capital of East Welega Zone. The town is located 328 km from Addis Ababa, the capital city of Ethiopia. The total population of Nekemte town is estimated to be 100,000. The estimated proportion of adolescents will be 25% of the total population. The town has one public university and many private colleges.

#### 2.1.1. Independent Variables

Age, sex, religion, educational status, marital status, family status, occupational status, and khat chewing are the independent variables.

#### 2.1.2. Dependent Variables

Prevalence of khat chewing and perceived psychological impact mainly depression and anxiety are the dependent variables.

#### 2.1.3. Source Population

Population of Nekemte town in adolescents and adults age group is the source population.

#### 2.1.4. Study Population

Those populations of Nekemte town in adolescents and adults age group are included in the study. For the in-depth interview, khat chewers in the same age range were the study populations.

### 2.2. Sample Size and Sampling Technique

#### 2.2.1. Sample Size

In calculation of sample size prevalence of khat and psychological impact have been considered and the larger sample size was taken. Sample size was calculated using single population proportion determination formula by considering a prevalence of khat chewing 30.6% from previous study [9], 95% confidence interval (*α* = 0.05), margin of error 5%, and 10% nonresponse rate; the final sample size is becoming 359. For economic and social impacts no adequate literature in the similar setting to calculate sample size quantitatively. Only qualitative sample is considered for those variables. For the qualitative data 15 in-depth interviews (IDIs) were conducted.

#### 2.2.2. Sampling Techniques

In order to obtain a more representative sample with highest precision, systematic random sampling was used. Since the town population is homogenous stratification and multistage sampling methods are not used to obtain the households. The sampling frame of the town household lists was obtained from the town administration and sampling fraction (*k*) was calculated and it was 10. The 1st sample household chosen from 1 to 10 by lottery method was 6 then the remaining possible sample households were chosen every 10 households. In those households having more than one eligible respondent, one was selected randomly by lottery method. In addition, purposive sampling was conducted to select khat chewers for IDIs.

### 2.3. Data Collection Methods, Instruments, and Collectors

For quantitative data, pretested and structured questionnaire was used and face-to-face interview was conducted by the selected and trained data collectors (B.S. holders). The structured questionnaire which was prepared in English and translated in to local language was used for data collection. As to the qualitative data, open ended in-depth interview guide was applied to explore profound information mainly on economic and social impacts of khat chewing. The findings from the qualitative data are triangulated with the quantitative findings to get strong evidences. Data collectors and supervisors were trained by principal investigators. The training was focused on objective of the study, confidentiality of information and the contents of the questionnaire in detail. Further training was also offered to supervisors on data quality management.

### 2.4. Data Quality Control

Pretest prior to applying to the sample was done on 5% of the sample size on those people who did not participate in the final data collection and some corrections were performed accordingly. Moreover, to maintain data quality, three days of intensive training was given for data collectors and for supervisors.

### 2.5. Data Processing and Analysis

Collected questionnaire was checked for completeness and consistency. Data was coded and entered into Epi Data version 3.1 statistical package software and it was exported to Statistical Package for Social Sciences (SPSS) software version 20.0 for analysis of descriptive statistics and statistical inferences. Both bivariate and multivariate logistic regression analysis was performed to quantify the effect of khat chewing on perceived psychological impacts as observed mainly by depression and anxiety. Test of statistical significant association was declared by using 95% CI and* p *value < 0.05.

Analysis of qualitative data is through thematic analysis: first the interview was typed up or transcribed and translated into English, then the text was read, and key themes were identified. Finally, the result is presented along with quantitative finding by using evidences from raw data as direct quotes (paraphrases).

### 2.6. Operational Definition

*Adolescents*. It is those respondents aged 15 to 24 years.

*Adults*. It is those study participants aged 25 and above years.

*Current Prevalence of Khat Chewing*. It is the proportion of the study population who were chewing khat within 30 days preceding the study.

*Depression and Anxiety State.* It was known based on* Diagnostic and Statistical Manual of Mental Disorders* 4th edition (DSM-IV) classification criteria [25].

Accordingly,* depression* is defined as experience of 4 or more of the following symptoms stated in DSM-IV for at least the past 6 months: loss of interest, trouble thinking, feeling unhappy/sad, crying more than usual, difficulty in enjoying (in life conditions), difficult decision making in life event, work suffering, not participating in useful/expected/event (like wedding, mourning conditions), feeling worthless/valueless, thought ending life (suicidal attempt), feeling tired, diminished appetite, pessimistic/hopeless/negative/views of the future, disturbed sleep, reduced self-esteem and self-confidence, reduced concentration and attention, and ideas of guilt.

*Anxiety.* It is defined as experience of 3 or more of the following symptoms stated in DSM-IV generalized anxiety disorder for at least the past 6 months: continuous feelings of nervousness (overacting), trembling, muscular tension (feeling of physical tension), sweating, cold and clammy hands, lightheadedness, palpitations (increased pulse rate at rest), dizziness, epigastria discomfort, and fears/worries.

### 2.7. Ethical Consideration

The proposal of this study was approved by Wollega University ethical review committee. Permission was also obtained from the concerned bodies of East Wollega Zonal Health Department and the responsible bodies of Nekemte town administration. An informed verbal consent was also obtained from the respondents.

## 3. Results

### 3.1. Sociodemographic Characteristics of the Study Participants

In this study the overall response rate was more than 98%. Out of the total study participants 292 (82.5%) were male, 215 (60.7%) were in age group 15–24 years, 123 (34.7%) were protestant in religion, 235 (66.4%) were single in terms of marriage, 179 (50.6%) have completed secondary education, and about 69 (19.5%) were unemployed (Table 1).

### 3.2. Current Chewing Status and Patterns among Study Participants

Out of all study participants 172 (48.6%) were found to be khat chewers in the last one month preceding the survey. Khat chewing experiences were higher among; male (89.5%) than female, age group 15–24 years (59.9%) than in any other age group, Muslims (44.2%) than any other religion follower, single (65.7%) than married as well as divorced/widowed respondents, secondary education completed respondents (47.7%) compared to respondents having other educational status and daily laborer respondents (22.1%) compared to respondents having other occupation (Table 1). The proportion of khat chewing practice within the group was higher among Muslim religion followers than other religion followers. This finding can be supported by in-depth interview of a 30-year-old man: “*when I was a child I used to attend a mosque and support older religious people. Usually those religious people were chewing chat and they also encourage us to chew khat. We respect those religious men and daily we tried chewing khat. Later I found myself as a regular chewer and adapted chewing khat as usual practices.” *

In the present study, among current chewers about 71 (41%) chew for more than 3 years, 116 (67%) chew daily, 74 (43%) smoke during chewing, 111 (64.5%) use coffee during chewing, and 65 (37.8%) drink alcohol after chewing (Table 2). Use of other substances along with khat chewing can be supported by in-depth interview of 27-year-old male: “*Chewing khat most likely increases the use of other substances. These substances may range from alcohol, cigarette, hashish, coffee to other drugs. These multiple use of substances affects the health and life of the users and their families. When multiple substances are used the effect seems unnoticed and the impact manifests in next day. Chewing khat pushes to use large doses of alcohols and other substances. The common term *‘*chebsi*'* implies such practices.*”

Moreover, the study found khat chewing practices were significantly associated with sex, religion, and marital status of the respondents. The odds of khat chewing practices was about 3 times higher (AOR = 3.23, 95% CI = 1.57, 6.64) among male respondents compared to female counterparts. The occurrence of khat chewing practices was about 3 times higher (AOR = 3.27, 95% CI = 1.85, 5.78) among Muslim religion followers and about 2 times higher (AOR = 2.07, 95% CI = 1.14, 3.77) among orthodox religion followers compared to those protestants. Lastly the odds of khat chewing practices were about 7 times higher (AOR = 6.85, 95% CI = 1.857, 25.287) among those respondents having divorced/widowed marital status compared to those unmarried respondents.

### 3.3. Factors Triggering for Khat Chewing Practices

In this study, various factors that initiate khat chewing were reported by current chewers. Accordingly 31 (18%) were chewing to confirm to their friends/family members, 44 (25.6%) to be relaxed, 53 (30.8%) to keep alert and concentration on work, 27 (15.7%) to spend extra time, 7 (4.1%) for praying and 10 (5.8%) due to being unemployed. The result can be supported by in-depth interview (IDI) of a 25-year-old male: “*The main triggering cause for starting chewing was being workless. As I think this is also the case in most young generations. If you don't have work where shall you go? If you look at many people around us here, many young people have found to be unemployed and almost all of them are forced to chew khat. It seems hard to find nonchewers in this village and the rate is also increasing.” *In-depth interview of 28- and 30-year-old male also showed the following:* “We chew khat because we didn't have work.” *Moreover In-depth interview of a 27-year-old male also showed the following:* “I adapted it from my friends. It increases excitement and alertness for work. When I chew khat I feel satisfied and happy. It is something that makes me free. For jobless people it lessens the mental strain they feel. Otherwise you will worry more for your life conditions.”*

### 3.4. Withdrawal Symptoms

The reported symptoms of khat chewers in time of lack of chewing practices include mood disturbance (low mood) by 42 (24.4%) of chewers, headache by 36 (20.9%), blurring of vision by 18 (10.5%), dizziness by 14 (8.1%), and irritability by 62 (36%) of chewers. This finding can be supported by in-depth interview of a 30-year-old male who chewed khat for 2 years of duration as “*When I didn't chew khat I feel irritable and restless. I feel completely bored in life and daily career. No focus and concentration thus I must find and chew khat, so that my emotion retain in my usual conditions I experienced.”* In-depth interview of a 27-year-old male respondent also showed the following: “*By the time I didn't chew khat, I didn't feel free. There is general malaise. For some people it hinders daily work. It also causes depression if ceased. People are forced to harm the society knowingly or unknowingly if khat is not chewed.”* Moreover in-depth interview of a 25-year-old male respondent showed the following: “*If khat is not chewed I feel sleeplessness, mood disturbance, irritability, negative feeling and discomfort. Psychologically khat chewing may change mood of the chewers. If you chew you may be excited and if you don't chew you will be in stress. You don't stay with your natural feelings.” *

### 3.5. Socioeconomic Impact

This study found that out of 172 who currently chew khat 52 (30.2%) had no adequate relationships with their family and about 45 (26.2%) had no participation in vital life events (like wedding, mourning conditions) with neighbors. Similarly, this study also found that, the average cost of khat for single consumption was 31 birr with ±21.4 SD and average cost of khat per month were 790 birr with ±710 SD. This expense on khat is more or less equivalent to a government salary for the lowest payable job in Ethiopia as well as daily payment of private organizations in the country. Almost all of the respondents participated in IDI said “*On average chewers could expense about 30 birr per single consumption of khat only. There can also be other associated costs occasionally.”*

### 3.6. Prevalence of Depression and Anxiety and Relationship with Khat Chewing Practices among Study Population

The present study found that the overall prevalence of depression and anxiety was 34.7% and 29.7%, respectively. In addition, about 36 (10.2%) study participants had complaints of hallucination, 31 (8.8%) had complaints of delusion, 31 (8.8%) showed sign of dependency for chewing and about 32 (9%) have showed sign of tolerance. Tendency to be dependent on khat chewing can be elaborated by IDI of a 30-year-old male as follows: “*Chewers mind are adapt to khat chewing and if they don't chew they are urged to find khat by paying whatever cost it had. Some people who are dependents may cheat and act in antisocial manner if they didn't have money to buy khat. Sometimes such practices are out of control of the individual.” *

Bivariate logistic regression analysis result found that the odds of depression among chewers were about 18 times higher (COR = 18.79, 95% CI = 10.19, 34.65) than those of nonchewers. Similarly this study found the odds of anxiety among chewers were about 5 times higher (COR = 5.09, 95% CI = 3.05, 8.51) than those of nonchewers. In multivariate logistic regression analysis result the occurrence of depression was about 25 times higher (AOR = 25.36, 95% CI = 12.13, 53.05) among khat chewers compared to nonchewers (Table 3). Similarly multivariate logistic regression analysis found the odds of anxiety among khat chewers were about 5 times higher (AOR = 5.49, 95% CI = 3.04, 9.96) compared to nonchewers (Table 4).

## 4. Discussions

The present study found that the current prevalence of khat chewing is 48.6%. The finding is lower than a study conducted in Butajira, Ethiopia [6]. The lower prevalence of the present study may be attributed to difference in study area which implies variability in terms of sociocultural value like norms and beliefs. In some society the khat chewing practice is believed to increase societies and relationships as people chew khat in ceremonial and work settings. In others khat is believed to be related with evil spirits and acts.

In this study khat chewing practices were found to be more prevalent among male than female which is consistent with previous local studies [9, 26]. This may be attributed to gender role and norm in the society that females are more responsible for caring of household members rather than expensing the money they earn for substance abuse like khat than males. The other possible justification could be underestimation of the prevalence of khat chewing in females as fewer females have participated in the study than males.

The proportion of khat chewing practice within the group was higher among Muslim religion followers than other religion followers which are supported by previous local studies [9, 26]. This may be attributed to the belief of Muslim khat chewers as khat chewing enhances concentration during prayer and enhances devotion. This may also be attributed to encouragement of chewing practices of younger age groups by some Muslim religious leaders and elder Muslim community.

Younger people attend to follow and support older religious people, if those religious people were chewing khat and the younger are encouraged to chew khat. This may be daily practice they try chewing khat. Later they found themselves as a regular chewer and adapted chewing khat as usual practice. Respected people in the society are the main influencers for khat chewing. In a certain community people want to confront with social practices and standards and respect the elders and religious people. So they are forced to practice chewing. But the respected people who train others chewing may not know the negative effect of khat chewing to the life of young people.

In this study the risk of khat chewing practices were significantly higher among divorced/widowed respondents. This may be due to the fact that the condition might affect the socioeconomic status of the family and heading the overall living condition of the family members by only father or mother might put him/her in worrying situation and mental stress that may intern leads to initiation of khat chewing to get temporary relief as well as to forgot the situation due to psychoactive stimulant effect of the leaves. In addition, the present study also found those divorced/widowed respondents were at risk of depression and anxiety compared to married and single respondents.

The study found routine khat chewing practices affect different aspects of the living condition of study participants. The influence can be categorized as social, economic, and psychological consequences.

Accordingly this study found khat chewing practices were significantly associated with marital status. In addition, about 30% of current khat chewers had no adequate relationship with their family and about 26% had no participation in vital life events with neighbors. This may be attributed to chewing khat causes irritability as well as chewers spend much of their time away from their houses thus warning the harmony of the family. Moreover, chewing practices may cause impairment on sexual intercourse which further leads to family instability.

In view of majority of community members chewers are less accepted and those people are tried to force the chewers to cease chewing. This social exclusion contributes for family disruption. In family life the expense for buying khat becomes the cause for conflict of the spouse with the husband as the husband expenses more money for khat chewing. The chewers also have no adequate time and care for their family. He/she forget his/her family.

It is known that khat chewing practices affects the economic status of the community due to the fact that, it leads to loss of working hours or absenteeism from work as well as utilization of money to buy the khat rather than expensing for buying nutritious foods and care of household members. In this regard, the present study found the mean time spent for single khat chewing ceremony was above 2 hours. In addition the average cost of khat for single consumption was 31 birr with ±21.4 SD and average cost of khat per month was 790 birr with ±710 SD.

The economic impact of khat chewing is losing of job or decreasing of productivity. This is common in long time chewers and dependent groups. The long time they use for chewing is in expense of the working time and chewers may be fired from employment or lose their private job.

In this study khat chewing practices were significantly associated with depression and anxiety which is in line with previous local studies conducted elsewhere [27, 28]. In addition khat chewing practices were significantly associated with hallucination and delusion. This could be due to psychoactive ingredients in khat like an amphetamine-like substance having psychomotor stimulant effects on central nervous system.

This study might have the following limitations. As the study was cross-sectional in nature it does not show cause-effect relationships meaning whether the reported and perceived psychological problems were caused by khat chewing practices or whether khat chewers were already suffering from the reported psychological problems by the time they started chewing. In addition because of interview nature of the study, it could offer a chance for interviewer bias. Moreover there is a chance of social desirability bias due to sensitive nature of the issue in some community members as it may enforce the respondents to deny any khat chewing practices.

## 5. Conclusions and Recommendations

In this study the current prevalence of khat chewing practices was 48.6%. Higher prevalence of khat chewing practices were observed among male sex, in younger age group, Muslim religion followers, unmarried respondents, secondary education completed and daily laborers. Khat chewing practices were significantly associated with sex, religion, and marital status of the respondents.

The study revealed khat chewing practices are leading to different socioeconomic problems as measured by family disruption, lack of adequate relationship with the family members, lack of participation in vital life events, time spent to chew khat, and money expensed to buy khat which is nonnutritious. More importantly, spending much money for buying khat might compromise distribution of resources for fulfillment of family members especially children's basic needs like food, clothes, shelters, home, education and others. In log run the condition might leads the family with excessive hunger; live in street (homeless); illiteracy, divorce etc. The occurrence of reported perceived psychological problems mainly depression and anxiety were significantly higher among khat chewers compared to nonchewers.

Based on the findings the following possible solutions are suggested.

- Organized efforts should be made to decrease the magnitude of the problem like the following.
  1. Creating awareness for the community of the study area about negative effect of khat chewing practices.
    - The town administrative bodies, regional health bureau, and other concerned and interested bodies should collaborate to mobilize the community and prepare different awareness creation teaching environments especially for the youths targeted at economic crisis of chewing practices; on the life of family members mainly on living condition of children, on marital status of the family, on living condition of the society.
  2. Particularly for younger age groups of the town making available of various methods of recreation in order to enable them to recreate on their free time.
  3. Preparing an educative communication forum with engagement of religious leaders, community leaders, health extension workers, the youths, adults, and concerned health planners to reach common understanding and to set common conventions (rules and regulations) that integrate the idea of different stalk holders regarding this substance use especially by young generation.
- Large scale follow-up study is required to prove the cause and effect relationships of khat chewing practices and mainly psychological impacts.
- Large scale qualitative study that engages religious leaders, community leaders, health professionals is suggested.

## Figures and Tables

**Table 1:** Sociodemographic characteristics of the study participants in Nekemte town, west Ethiopia, 2016.

Variables	Characteristics	Chewers (%)	Nonchewers (%)	Total (%)
Sex of participants	Male	154 (89.5%)	138 (75.8%)	292 (82.5%)
	Female	18 (10.5%)	44 (24.2%)	62 (17.5%)

Age of participants in year	15–24	103 (59.9%)	112 (61.5%)	215 (60.7%)
	25–34	61 (35.5%)	65 (35.7%)	126 (35.6%)
	≥35	8 (4.7%)	5 (2.7%)	13 (3.7%)

Religion of participants	Protestant	39 (22.7%)	84 (46.2%)	123 (34.7%)
	Muslim	76 (44.2%)	47 (25.8%)	123 (34.7%)
	Orthodox	52 (30.2%)	47 (25.8%)	99 (28.0%)
	Others^1^	5 (2.9%)	4 (2.2%)	9 (2.5%)

Marital status	Single	113 (65.7%)	122 (67.0%)	235 (66.4%)
	Married	41 (23.8%)	56 (30.8%)	97 (27.4%)
	Divorced/widowed	18 (10.5%)	4 (2.2%)	22 (6.2%)

Educational status	Can read and write	8 (4.7%)	2 (1.1%)	10 (2.8%)
	Primary	53 (30.8%)	41 (22.5%)	94 (26.6%)
	Secondary	82 (47.7%)	97 (53.3%)	179 (50.6%)
	Higher education	29 (16.9%)	42 (23.1%)	71 (20.1%)

Occupational status	Merchant	37 (21.5%)	33 (18.1%)	70 (19.8%)
	Employed	35 (20.3%)	38 (20.9%)	73 (20.6%)
	Daily laborer	38 (22.1%)	31 (17.0%)	69 (19.5%)
	Student	29 (16.9%)	44 (24.2%)	73 (20.6%)
	Unemployed	33 (19.2%)	36 (19.8%)	69 (19.5%)

^1^Catholic, no religion.

**Table 2:** Current khat chewing status and patterns among the study participants in Nekemte town, west Ethiopia, 2016.

Variables	Characteristics	Frequency (%)
Current chewing experience	Nonchewers	182 (51.4)
	Chewers	172 (48.6)

Chewing duration	≤12 months	48 (27.9)
	13–36 months	53 (30.8)
	>36 months	71 (41.3)

Chewing frequency	Daily	116 (67.4)
	4–6 times a week	31 (18.0)
	2-3 times a week	16 (9.3)
	Once a week	9 (5.2)

Time spent on single chewing ceremonies	15–90 minutes	58 (33.7)
	120–180 minutes	85 (49.4)
	>180 minutes	29 (16.9)

Cost of khat for single consumption	5–15 birr	38 (22.1)
	16–25 birr	52 (30.2)
	26–50 birr	65 (37.8)
	>50 birr	17 (9.9)

Cost of khat per month	10–100 birr	14 (8.1)
	101–400 birr	33 (19.2)
	401–600 birr	54 (31.4)
	601–900 birr	33 (19.2)
	≥901 birr	38 (22.1)

Sugar use during chewing	Yes	139 (80.8)
	No	33 (19.2)

Smoking during chewing	Yes	74 (43)
	No	98 (57)

Coffee use during chewing	Yes	111 (64.5)
	No	61 (35.5)

Alcohol use after chewing	Yes	65 (37.8)
	No	107 (62.2)

**Table 3:** Bivariate and multivariate logistic regression analysis result which shows mainly the association between khat chewing practices and depression in Nekemte town, west Ethiopia, 2016.

Variables	Characteristics	Depression number (%)	COR (95% CI)	AOR (95% CI)
Current chewing experiences	Nonchewers	15 (8.2%)	1	1
	Chewers	108 (62.8%)	18.79 (10.19, 34.65)^*∗*^	25.36 (12.13, 53.05)^*∗*^

Sex of participants	Male	110 (37.7%)	2.28 (1.18, 4.39)^*∗∗*^	1.75 (.64, 4.79)
	Female	13 (21.0%)	1	1

Age of participants in year	15–24	59 (27.4%)	1	1
	25–34	56 (44.4%)	2.12 (1.33, 3.36)^*∗∗∗*^	3.73 (1.74, 7.99)^*∗∗∗*^
	≥35	8 (61.5%)	4.23 (1.33, 13.45)^*∗∗*^	4.83 (.83, 28.09)

Religion of participants	Protestant	30 (24.4%)	1	1
	Muslim	41 (33.3%)	1.55 (.89, 2.71)	.737 (.34, 1.58)
	Orthodox	47 (47.5%)	2.80 (1.58, 4.96)^*∗*^	2.589 (1.17, 5.72)^*∗∗*^
	Others^1^	5 (55.6%)	3.88 (.98, 15.37)	2.69 (.39, 18.66)

Marital status	Single	76 (32.3%)	.18 (.07, .48)^*∗∗∗*^	.53 (.14, 2.07)
	Married	31 (32.0%)	.18 (.06, .49)^*∗∗∗*^	.35 (.09, 1.33)
	Divorced/widowed	16 (72.7%)	1	1

Educational status	Can read and write	8 (80.0%)	1	1
	Primary	34 (36.2%)	.14 (.03, .71)^*∗∗*^	.14 (.02, 1.12)
	Secondary	54 (30.2%)	.11 (.02, .53)^*∗∗*^	.17 (.02, 1.33)
	Higher education	27 (38.0%)	.15 (.03, .78)^*∗∗*^	.20 (.02, 1.75)

Occupational status	Merchant	24 (34.3%)	1	1
	Employed	30 (41.1%)	1.34 (.68, 2.64)	1.41 (.53, 3.79)
	Daily laborer	26 (37.7%)	1.16 (.58, 2.32)	1.19 (.45, 3.14)
	Student	15 (20.5%)	.50 (.23, 1.05)	.90 (.32, 2.55)
	Unemployed	28 (40.6%)	1.31 (.66, 2.61)	1.99 (.72, 5.53)

^*∗*^ *p* = 0.000; ^*∗∗*^*p* < 0.05; ^*∗∗∗*^*p* < 0.005; ^1^Catholic, no religion; COR = crude odds ratio; AOR = adjusted odds ratio; CI = confidence interval.

**Table 4:** Bivariate and multivariate logistic regression analysis result which shows mainly the association between khat chewing practices and anxiety in Nekemte town, west Ethiopia, 2016.

Variables	Characteristics	Anxiety number (%)	COR (95% CI)	AOR (95% CI)
Current chewing experiences	Nonchewers	26 (14.3%)	1	1
	Chewers	79 (45.9%)	5.09 (3.05, 8.51)^*∗*^	5.49 (3.04, 9.96)^*∗*^

Sex of participants	Male	88 (30.1%)	1.14 (.62, 2.10)	1.19 (.54, 2.63)
	Female	17 (27.4%)	1	1

Age of participants in year	15–24	50 (23.3%)	1	1
	25–34	48 (38.1%)	2.03 (1.26, 3.28)^*∗∗∗*^	2.03 (1.07, 3.84)^*∗∗*^
	≥35	7 (53.8%)	3.85 (1.24, 11.98)^*∗∗*^	3.26 (.77, 13.85)

Religion of participants	Protestant	30 (24.4%)	1	1
	Muslim	36 (29.3%)	1.28 (.73, 2.26)	.79 (.39, 1.55)
	Orthodox	35 (35.4%)	1.69 (.95, 3.04)	1.18 (.59, 2.34)
	Others^1^	4 (44.4%)	2.48 (.63, 9.84)	1.66 (.34, 8.04)

Marital status	Single	56 (23.8%)	.09 (.032, .26)^*∗*^	.17 (.05, .58)^*∗∗∗*^
	Married	32 (33.0%)	.15 (.05, .43)^*∗*^	.30 (.09, .99)^*∗∗*^
	Divorced/widowed	17 (77.3%)	1	1

Educational status	Can read and write	4 (40.0%)	1	1
	Primary	31 (33.0%)	.74 (.19, 2.81)	1.33 (.25, 7.07)
	Secondary	51 (28.5%)	.59 (.16, 2.21)	1.44 (.28, 7.44)
	Higher education	19 (26.8%)	.55 (.14, 2.16)	.79 (.14, 4.53)

Occupational status	Merchant	19 (27.1%)	1	1
	Employed	26 (35.6%)	1.49 (.73, 3.03)	1.95 (.79, 4.80)
	Daily laborer	18 (26.1%)	.95 (.45, 2.01)	1.39 (.56, 3.43)
	Student	17 (23.3%)	.82 (.38, 1.74)	2.04 (.79, 5.21)
	Unemployed	25 (36.2%)	1.53 (.74, 3.13)	3.05 (1.22, 7.64)^*∗∗*^

^*∗*^ *p* = 0.000; ^*∗∗*^*p* < 0.05; ^*∗∗∗*^*p* < 0.005; ^1^Catholic, no religion; COR = crude odds ratio; AOR = adjusted odds ratio; CI = confidence interval.
